# Comparison of tracer kinetic models in differentiating malignant from normal prostate tissue using dynamic contrast-enhanced MRI

**DOI:** 10.3389/fonc.2024.1450388

**Published:** 2024-12-06

**Authors:** Hongjiang Zhang, Jing Yang, Kunhua Wu, Zujun Hou, Ji Du, Jianhua Yan, Ying Zhao

**Affiliations:** ^1^ Department of Magnetic Resonance Imaging (MRI), The First People’s Hospital of Yunnan Province, The Affiliated Hospital of Kunming University of Science and Technology, Kunming, China; ^2^ Department of Radiology, FISCA Laboratory for Advanced Imaging, Nanjing, China; ^3^ Department of Nuclear Medicine, The First Affiliated Hospital of University of Science and Technology of China, Hefei, Anhui, China

**Keywords:** DCE MRI, tracer kinetic modeling, prostate cancer, quantitative, Gleason score

## Abstract

**Purpose:**

The aim of this study was to evaluate the diagnostic value of dynamic contrast-enhanced magnetic resonance imaging (DCE-MRI) derived kinetic parameters with high spatiotemporal resolution in discriminating malignant from normal prostate tissues.

**Methods:**

Fifty patients with suspicious of malignant diseases in prostate were included in this study. Regions of interest (ROI) were manually delineated by experienced radiologists. Voxel-wise kinetic parameters were produced with the following tracer kinetic models (TKMs): Tofts model, extended Tofts model (ETM), Brix’s conventional two-compartment model (Brix), adiabatic tissue homogeneity model (ATH), and distributed parameter model (DP). The initial area under the signal-time curve (IAUC) with an uptake integral approach was also included. Mann–Whitney U test and receiver operating characteristic (ROC) curves were used to evaluate the capability of distinguishing tumor lesions from normal tissues. A p-value of 0.05 or less is considered statistically significant. ROI based parameters correlation analysis between DP and ETM were performed.

**Results:**

624 lesions and 269 normal tissue ROIs were obtained. Thirty parameters were derived from the six kinetic models. Except for PS from Brix, statistically significant differences between lesions and normal tissues (P<0.05) were observed in other parameters.Ve from DP, ATH and Brix and PS from ATH have AUC values less than 0.6 in the ROC analysis. MTT, Vp and PS from DP, Ktrans from ETM and Tofts, E and PS from ATH, IAUC parameters and F from Brix have AUC values larger than 0.8. Ve and Vp from DP and ETM are correlated (r> 0.65). The correlation coefficient between Ktrans from ETM and PS from DP is 0.751.

**Conclusion:**

MTT, Vp and PS from DP, Ktrans from ETM and Tofts, E and PS from ATH, F from Brix and IAUC parameters can be used to differentiate malignant lesions from normal tissues in the prostate.

## 1 Introduction

Prostate cancer (PCa) ranks sixth in incidence and seventh in cancer-related mortality rates in China (1).In 2020, approximately 1.4 million new cases of PCa were diagnosed worldwide. The Lancet Commission predicts that by 2040, the incidence rate will double, with an estimated 3 million new cases (2). Magnetic resonance imaging (MRI) is becoming an important method in the diagnosis and management of PCa, Its strong negative predictive value is instrumental in mitigating the risks of overdiagnosis and overtreatment of clinically significant prostate cancer(csPCa) (3, 4) and MRI-guided biopsy is associated with higher detection rates (5). In 2019, the Prostate Imaging Reporting and Data System (PI-RADS) steering committee released PI-RADS version 2.1 (PI-RADS v2.1) by revising acquisition parameters and the scoring system in the PI-RADS version 2 while maintaining the overall framework of the system (6). DCE-MRI is the rapid acquisition of sequential images during the passage of a contrast agent within a tissue of interest over a period, which potentially provides information regarding tumor angiogenesis such as blood flow,vascular permeability, and micro-vessel density to aid treatment selection, frequent treatment monitoring and assess response to targeted therapy following treatment. The clinical application of DCE-MRI in PCa management is supported by evidence that malignant lesions enhance and wash out more quickly than normal prostate tissues (2).

In the PI-RADS v2.1, DCE-MRIis mandated to be interpreted in conjunction with T2-weighted imaging(T2WI) and diffusion weighted imaging(DWI), both of which have greater weight and influence in assessment. The role of DCE-MRI in PI-RADS v2.1 has been downgraded to a qualitative binary classifier in the lesion within the peripheral zone of the prostate only when differentiating between a PI-RADS score of 3 and 4. However, only qualitative (uptake and washout curve pattern with limited imaging time points) or semi-quantitative methodology was reviewed in PI-RADS v2.1. The downgrading of DCE-MRI in the PI-RADS guidelines could possibly be related to variation in DCE data acquisition and analysis, where visual examination by radiologists was the dominant method for DCE image analysis. Using the visual review approach, Lotte et al. reported that the accuracy of mpMRI was not significantly improved by adding DCE to T2WI and DWI (7), whereas Zawaideh et al. reviewed two hundred sixty-four patients who underwent prostate MRI, and found that mpMRI had fewer Likert 3 call rates and increased specificity and was subjectively considered of benefit by readers in 28.4% of cases (8).

Verma et al. presented a comprehensive review on the application of DCE-MRI to PCa diagnosis, where the analysis of prostate DCE-MRI can be categorized into three types of methods: qualitative, semi-quantitative, and quantitative (9). Qualitative analysis visually assess whether focal areas enhance earlier and more intensely than normal tissues, which is inherently subjective and varies in different prostate zones. Semi-quantitative analysis evaluate the shape of the intensity-time curve in the region of interest (ROI) by calculating various curve parameters include the time of first contrast uptake, time to peak, maximum slope, and peak enhancement, which is sometimes collectively referred to as “curveology.” (9). Tavakoli et al. recently investigated the contribution of mean early-phase DCE signal (mDCE) to PI-RADS for detecting csPCa and found that mDCE did not assist with PI-RADS score 3 lesion risk stratification (10).

The quantitative approach is based on modeling the transport of the contrast agent within the tissue microenvironment using tracer kinetic modeling(TKM) techniques. well-known tracer kinetic models like Tofts (11) and extended Tofts(ETM) (12)have been extensively studied in PCa (9, 13). Winkel et al. assessed whether incorporating perfusion information from Tofts into T2WI/DWI sequences improved machine learning classification of PCa risk groups, discovering that this approach enhanced risk stratification for both benign and malignant, and intermediate- versus high-risk PCa in the peripheral zone (14). Park et al. evaluated the efficacy of ETM-derived perfusion parameters in distinguishing csPCa(Gleason score ≥7) from clinically insignificant PCa [(ciPCa) Gleason score 6], finding that the interstitial space volume fraction was most effective, albeit with a modest AUC of 0.643 (15).

Stabile et al. recently reviewed the factors affecting the accuracy of mpMRI and MRI-targeted biopsy to detect and localize csPCa, and found that the radiologists’ experience was the dominating factor (16). The high heterogeneity across the studies underlines the need to define the experience of radiologists and urologists, implement quality control, and adhere to the most recent PI-RADS assessment guidelines. Nevertheless, a factor that was not accounted in the review is the way how DCE images were acquired and analyzed. Among the studies included, most used the qualitative approach. DCE imaging has progressed tremendously along with more advanced TKMs being proposed (such as Brix’s conventional two-compartment model (17), adiabatic tissue homogeneity model (ATH) (18, 19), and distributed parameter (DP) model (20, 21), but with less attention in PCa diagnosis. Hence, further research is required to clarify whether technical advancement would impact the accuracy of the PCa MRI pathway and to what extent.

## 2 Materials and methods

### 2.1 Patient

This study received approval on June 28, 2021, with the approval number KHLL 2021-137 from the institutional research ethics review board of The First People’s Hospital of Yunnan Province, The Affiliated Hospital of Kunming University of Science and Technology, Kunming, China. A total of 72 consecutive patients diagnosed with PCa underwent a DCE-MRI examination between June 2022 and February 2024. The inclusion criteria were as follows: (a) patients with an elevated PSA; (b) patients with clinical symptoms; (c) no history of androgen castration treatment, chemoradiotherapy, or biopsy before MRI examination; (d) acceptable quality of MR images; and (e) pathological results obtained by a combination of standard transrectal ultrasound (TRUS)-guided 12-core systematic biopsy and MRI–TRUS cognitive fusion biopsy(n=33) or radical prostatectomy(n=17) within a week after the MR examination. Patients were excluded for the following reasons: (a) unsatisfactory image quality of DCE-MRI, such as significant motion artifacts(n=3) and difficulty in delineating artery input(n=10); (b) prostate biopsy performed within six weeks before the MRI examination(n=2); and (c) absence of histopathologic reports due to no biopsy or surgery was performed (n=7).

### 2.2 Image acquisition

All scans were performed on a 3.0T MRI scanner (MAGNETOM Prisma, Siemens Healthineers, Germany) in The First People’s Hospital of Yunnan Province, The Affiliated Hospital of Kunming University of Science and Technology, Kunming, China. Each scan included axial T1-weighted imaging (T1WI) (repetition time (TR)=500ms, echo time(TE)=9.7ms, slice thickness=3.0mm, gap=0.3mm, number of slices=24), T2WI in three planes(TR=3000-4000ms, TE=107-114ms, slice thickness=3.0mm, gap=0.3mm), DWI(TR=4000ms, TE=57ms,slice thickness=3.0mm, gap=0.3mm, 3 b-values:50,1000,2000s/mm^2^) and DCE-MRI. The DCE-MRI was performed using a three-dimension volumetric interpolated breath-hold examination (3D Vibe) sequence in the axial direction (TR= 2.8ms, TE=0.82ms; slice thickness=3.0mm, gap=0.6mm, FOV=300x248mm, matrix=160x99, number of slices=24, NEX= 1, pre-contrast flip angles: 5°, 10°, 15°, post-contrast flip angle 15°). Before injection of the contrast agent, ten repetitions of the sequence were performed for each flip angle (5°, 10°, 15°), and native (pre-contrast) tissue T1 values were estimated using the variable flip angle method. 120 dynamic scans with a temporal resolution of 2s were performed immediately after intravenous administration of Gadopentetate Dimeglumine(Magnevist; BayerSchering Pharma AG) at a rate of 2.0 mL/s and a dose of 0.1mmoL/kg body weight. Tissue contrast concentration-time curves were derived from the dynamic scans by estimating the difference in post- and pre-contrast relaxation rate(1/T1) for kinetic modeling.

### 2.3 Image analysis

DCE-MR image analysis were performed on a commercially available software (FISCA Healthcare, China). ROI in the foci that are suspicious of malignancy and normal tissue were manually demarcated by two experienced radiologists (with six and eighteen years of expertise in prostate MRI, respectively) on the artery phase of DCE images with cross-referencing of the T2WI, DWI and DCE-MRI scans. The arterial input function (AIF) for each subject was sampled from iliac artery. In total,6 parameters from DP, 3 parameters from Tofts, 4 parameters from ETM, 6 parameters from Brix, 7 parameters from ATH and 4 parameters from IAUC on a voxel-wise level were produced for each subject. Voxels that failed in fitting or with non-physiological values were excluded from further analysis. The parameters’ abbreviation, definition, and unit are listed in **Table 1**.

**Table 1:** Tracer kinetic parameters abbreviation, definition, and unit.

Parameters	Definition	Unit
F	Flowrateofwholebloodthroughthevascularcompartment	mL/min/mL
MTT	Meantransit time	sec
E	Initial (first-pass)extraction fraction	
Ktrans	Transferconstant	min^-1^
Ke	Effluxrateconstant	min^-1^
PS	Endothelialpermeabilitysurfaceareaproduct	mL/min/mL
Ve	Ratioof extra-vascularvolumetotissue volume	%
Vp	Ratioofbloodplasmavolumetotissuevolume	%
IAUC60	Initial area under the curve for first 60 seconds	
IAUC90	Initial area under the curve for first 90 seconds	
IAUC60No	IAUC60 in the lesion divided by IAUC60 in the aorta	
IAUC90No	IAUC90 in the lesion divided by IAUC90 in the aorta	

### 2.4 Statistical analysis

The Mann–Whitney U test was used to compare all parameter values in lesions that are suspicious of malignancy and normal prostate tissue. Spearman correlation analysis was used to assess possible correlations of parameters between DP and the commonly used ETM. A strong correlation was assumed for 0.8<r ≤ 1, a moderate correlation for 0.5<r ≤ 0.8, a weak correlation for 0.3<r ≤ 0.5 and no correlation for r ≤ 0.3 (22). Receiver operating characteristic (ROC) analysis was conducted to evaluate the discriminatory ability of each parameter, with the area under curve (AUC) serving as the indicator of discriminatory power. Optimal cut-off values were chosen using the Youden index on the estimated curves. An AUC value greater than 0.8 was considered to be effective in discriminating tumors from normal tissue. All statistical analyses were performed using MATLAB (2020b; MathWorks, Natick, MA) software. A p-value of 0.05 or less was considered to be statistically significant.

## 3 Results

### 3.1 Patients

**Figure 1:**
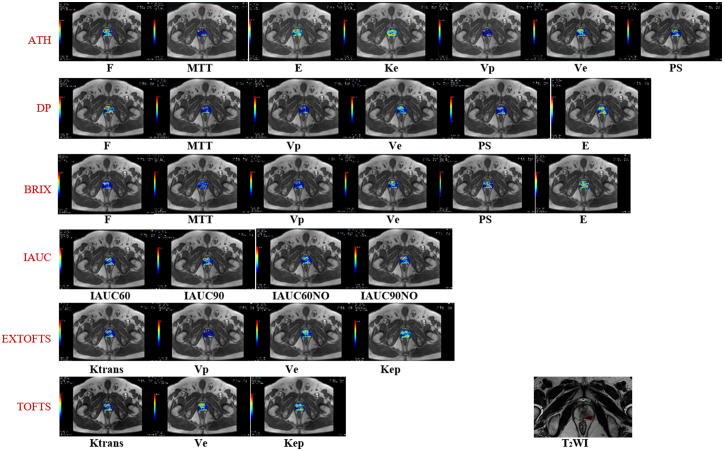
Shows representative parameter maps generated by using the various kinetic models. The corresponding T2 weighted image with a tumor ROI delineation.

Of the 72 cases, 50 were finally selected after exclusion (13 cases due to unsatisfactory image quality of DCE-MRI; 2 cases due to prostate biopsy performed within six weeks before MRI examination; 7 cases due to no histopathologic reports). Clinical information of the 50 patients is shown in **Table 2**. **Figure 1** shows representative parameter images of a patient generated by using the various kinetic models. A tumor ROI is shown in the T2WI. The tumor can be well visualized in most kinetic images.

**Table 2:** Demographic information of the patients included.

Patient Characteristics	
Variables	Value
Age (y)	
Median (range)	71 (67–77)
Prebiopsy PSA level (ng/mL)	
Median (range)	23.96 (12.91-76.87)
Prebiopsy PSA density (ng/mL)	
Median (range)	0.57 (0.30-1.82)
PI-RADS score	
1	0 (0)
2	0 (0)
3	4 (6)
4	20 (33)
5	37 (61)
Gleason score	
6	7 (11)
7	18 (30)
8	16 (26)
9	15 (25)
10	5 (8)
GGG	
1	7 (11)
2	8 (13)
3	11 (18)
4	15 (25)
5	20 (33)
csPCa	
Yes	64 (89)
No	7 (11)

PSA, prostate-specific antigen; PI-RADS, Prostate Imaging Reporting and Data System; GGG, Gleason grade group; csPCa, clinically significant prostate cancer.

### 3.2 Comparison of kinetic parameters and ROC analysis

**Figure 2:**
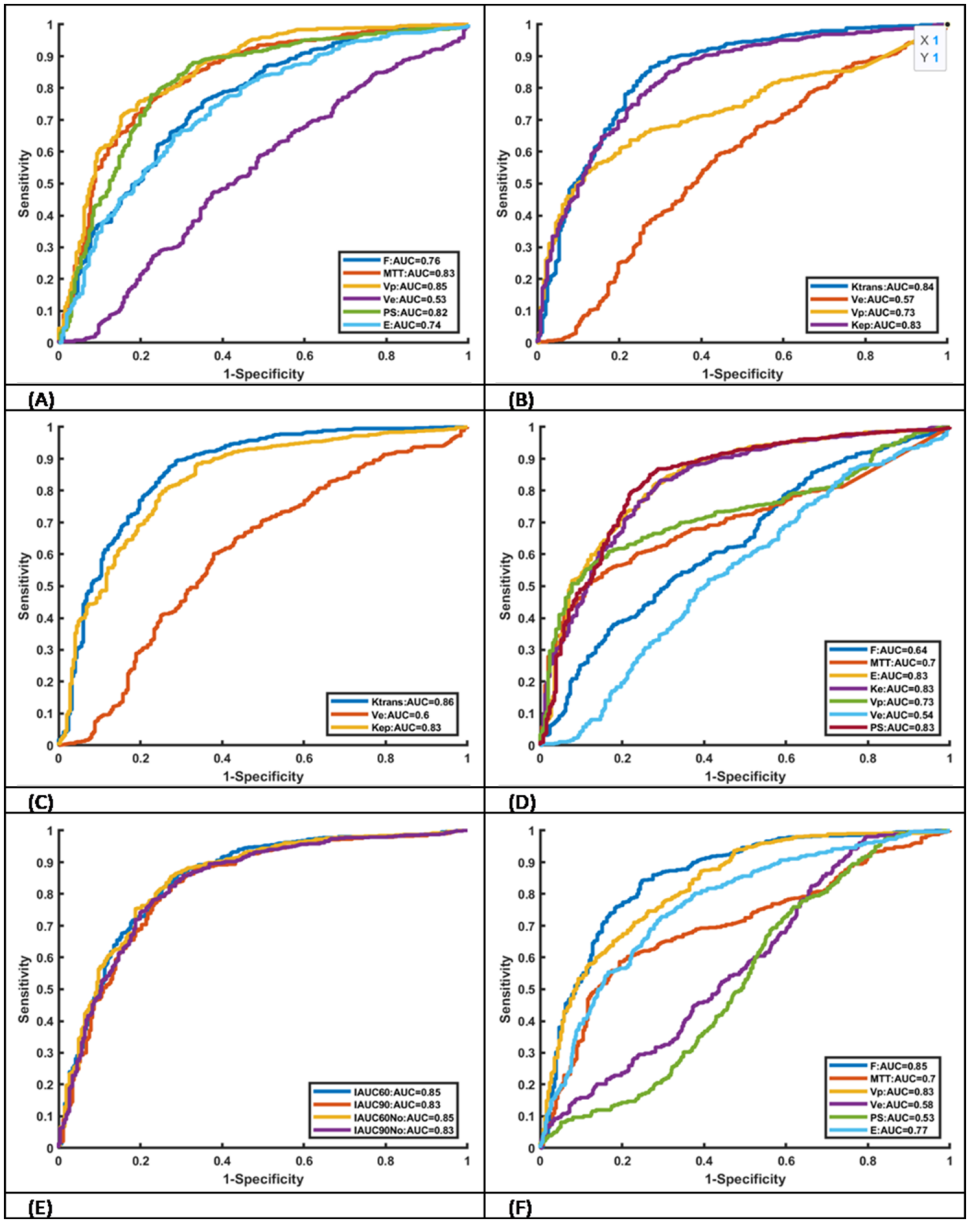
Receiver operating characteristic curves of kinetic parameters derived from six different models AUC values. **(A)** DP, **(B)** ETM. **(C)** TOFTS, **(D)** ATH, **(E)** IAUC and **(F)** Brix.

624 lesion ROIs and 269 normal tissue ROIs were identified. Median values for each parameter in lesions that are suspicious of malignancy and normal tissues are detailed in **Table 3**. Only PS from Brix shows no statistically significant difference between lesions that are suspicious of malignancy and normal tissues via the Mann–Whitney U test. All parameter values in lesions that are suspicious of malignancy are larger than those in normal tissues. The ROC curves of the various kinetic parameters with AUC values and thresholds are shown in **Figure 2**. MTT, Vp and PS from DP, Ktrans and Kep from ETM, Ktrans from Tofts, E and PS from ATH, F and Vp from Brix and IAUC parameters yielded large AUC values (>0.8) in discriminating tumor from normal tissue. Ve from all models exhibited poor performance with an AUC value of less than 0.6. Additionally, AUC value of PS from Brix is less than 0.6.**Table 4** summarizes the ROC curve analysis results including optimal cutoff value, sensitivity, specificity, and accuracy. Ktrans derived from the Tofts model gave the best performance in differentiating suspicious malignant lesions from normal prostate tissues (AUC=0.86), with a sensitivity of 83.9% and a specificity of 75.4% with a cutoff value of 0.07.

**Table 3:** Median parameter values in lesions and normal tissues derived from different kinetic models.

Lesion/Normal							
ATH	F 32.60/26.15	MTT 1.28/0.23	E 27.09/11.09	Ke 0.95/0.43	Vp 1.05/0.09	Ve 10.32/8.81	PS ** 11.57/3.83
DP	F 20.01/12.1	MTT 5.28/1.27	Vp 2.18/0.32	Ve 9.36/8.20	PS 11.63/4.19	E 43.41/26.32	
Brix	F 25.46/8.55	MTT 10.7/2.53	Vp 4.11/0.51	Ve 6.60/7.73	PS 4.32/4.58	E 14.65/36.39	
IAUC	IAUC60 4.26/1.47	IAUC90 7.42/2.78	IAUC60No 8.34/2.98	IAUC90No 10.70/4.21			
ETM	Ktrans 0.12/0.04	Vp 0.79/0.04	Ve 11.44/9.19	Kep 1.00/0.39			
Tofts	Ktrans 0.15/0.04	Ve 12.61/9.16	Kep 1.10/0.40				

Entry with P>0.05 are indicated by** based on Mann–Whitney U test between prostate cancer and normal tissue.

**Table 4:** Spearman’s correlation coefficient between parameters of DP model (F, tp, Vp, Ve, PS and E) and ETM (Ktrans, Ve, Vp and Kep) in lesions and normal tissues.

Lesions	Ktrans	Ve	Vp	Kep	Normal Tissues	Ktrans	Ve	Vp	Kep
F	0.656 **	0.246 **	0.442 **	0.112 **	F	0.439 **	0.060	0.605 **	0.297 **
MTT	0.382 **	0.174 **	0.563 **	0.057	MTT	0.518 **	0.291 **	0.351 **	0.323 **
Vp	0.644 **	0.289 **	0.675 **	0.104 **	Vp	0.773 **	0.180 **	0.681 **	0.509 **
Ve	0.017	0.916 **	0.078	-0.218 **	Ve	0.062	0.917 **	0.040	-0.173 **
PS	0.751 **	0.264 **	0.062	0.120 **	PS	0.361 **	0.351 **	0.137 **	0.133 **
E	0.424 **	0.209 **	-0.108 **	0.025	E	0.455 **	0.290 **	0.060	0.157 **

Entries with P<0.05 are indicated by**.

### 3.3 Correlations between DP and ETM parameters

The results of Spearman’s correlation coefficients between parameters of DP model are shown in **Table 4**. Except for the correlation between MTT and Kep, Ve and Ktrans, E and Kep in the lesions, Ve and Vp, E and Vp in the normal tissues, other correlations are statistically correlated with different coefficient values (P<0.05). Ve from DP is negatively correlated with Kep from ETM for both lesions and normal tissues. Ve from DP are highly correlated with those from ETM with correlation coefficient larger than 0.9. For lesions, both F and PS are positively correlated with Ktrans from ETM.

## 4 Discussion

In this study, quantitative DCE parameters from different TKMs were investigated to discriminate PCa and normal tissue. Results showed that (1) Vp from DP, F from Brix, Ktrans from Tofts and IAUC60 gave AUC values>0.85 (2), most parameters showed significant differences, and (3) all TKMs presented good performance, with one or more parameters AUC>0.80. All advanced TKMs demonstrated that prostate cancer tissue was characterized by higher values of F, Vp, MTT, PS and E in comparison with normal tissue. Except for Ktrans from Tofts, Vp from DP, F from Brix, IAUC60 and IAUC60No from IAUC, they also have excellent performance in discrimination with an AUC of 0.85. In this study, Ktrans from Tofts, which determines the rate of gadolinium influx from plasma into the extravascular extracellular space (EES), gave the best performance in terms of AUC value. Ktrans can describe only PS when the transport of the tracer across the vessel wall is limited by permeability in the situation of high blood flow. In the Mann–Whitney U tests, only PS from Brix did not give statistically significant differences between oncological lesions and normal tissues. Except for PS from Brix, PS from DP and ATH give high AUC values (larger than 0.8). This could be partially explained by the difference Brix and DP or ATH on the assumption of intravascular distribution of gadolinium contrast. In the ROC analysis, Ve from all TKMs, measuring the fractional volume of the EES, showed poor capability in differentiation, which suggests no significant difference of “room” available within the tissue interstitium for accumulating gadolinium between oncological lesions and normal tissues. correlation analysis between DP and ETM illustrated a moderately positive correlation between DP-derived PS and ETM-derived Ktrans in prostate cancer tissue, but a weakly positive correlation in prostate normal tissue.

For DCE imaging, 3D T1w-GRE with a temporal resolution of less than 15 seconds is recommended in PI-RADS v2.1 (6).The present study used a DCE-MRI protocol with a temporal resolution of 2 seconds, which may help capture rapid changes of signal intensity and improve quantitative analysis. However, short-interval image acquisition may easily lead to motion error. A robust image registration method dedicated to DCE kinetic modeling is being developed and will be incorporated in the image analysis protocol.

The presence of benign disease (e.g, benign prostatic hyperplasia, inflammation, prostatic intraepithelial neoplasia, and atrophy) is a common cause of false positive errors in diagnosing PCa using mpMRI. To determine the best features to discriminate PCa from benign disease and its relationship to benign disease classification and tumor grade, Litjens and colleagues derived a series of features from T2WI, ADC map, and DCE parametric maps (including Tofts-derived parametric maps), and used sequential forward floating feature selection analysis to identify the best combination of MRI parameters to discriminate among benign classes (23). The results showed that Ve maps provided the best separation between cancer and atrophy. For the other types of noncancerous lesions, features from all three modalities were included, indicating that each parameter in mpMRI provided additional information to the diagnostic process (23). The finding supported the added value of quantitative DCE in the characterization of nonmalignant lesions.

Akin and coauthors assessed the incremental value of DWI and DCE to T2WI in detecting locally recurrent PCa after radiotherapy, and they found that biopsy-positive and biopsy-negative prostate sides differed significantly in Tofts-derived Ktrans and Kep (24). Verma et al. presented a review of earlier efforts of DCE-MRI in prostate management and concluded that DCE-MRI was emerging as a useful clinical technique as part of a multi-parametric approach for evaluating the extent of primary and recurrent prostate cancer, however, performing a high-quality DCE-MRI examination required an in-depth knowledge of the technical aspects and limitations of image acquisition and postprocessing techniques (9). An appraisal of the application of mpMRI to PCa diagnosis was recently reported (16). Even though the use of DCE is currently of debate, DCE seems to be particularly useful when T2WI and DWI are equivocal or degraded by artefacts. In particular, DCE has demonstrated an important role in the evaluation of local recurrence after interventions (such as transurethral resection of the prostate and focal therapy) that change prostate morphology, where the standard PI-RADS score is not applicable (16). The value of DCE in detecting local PCa recurrence with biochemical relapse after local treatment with curative intent was recognized in the recently published Prostate Imaging for Recurrence Reporting (PI-RR) system (25), where the PI-RR assessment after radiation therapy is mainly derived from the DWI and DCE sequences (where DCE would be of particular importance when DWI could be subject to susceptibility artefacts after low-dose-rate brachytherapy), and the final PI-RR assessment score after radical prostatectomy is generated using the individual DWI and DCE sequences, with DCE being the dominant sequence. The long-term goal of this study is to explore whether quantitative kinetic parameters from dynamic scans can be used to improve the weighting of DCE imaging in the PI-RADS and PI-RR. Multi-center and multi-nation large-scale clinical trials are needed to address this issue.

There are several avenues to improve the study. Firstly, this is a single institution study and the size of the effective dataset is moderate, and the findings need to be further validated in multicenter trials with large cohort size. Secondly, lesions and normal tissues ROIs were manually delineated by a senior radiologist with more than 10 years’ experience, but this still may introduce variability of ROI delineation. Thirdly, this study does not differentiate the location of malignant lesions. In clinical practice, localized prostate cancer shows great clinical, genetic and environmental heterogeneity, and spatial distribution in tumorigenesis are being increasingly considered for further personalized treatment. Fourthly, this study does not investigate the difference between csPCa (Gleason score>6) and ciPCa (Gleason score=6), which could be studied in the future when more samples of ciPCa are recruited.

## 5 Conclusion

This study compared quantitative DCE parameters from different TKMs in distinguishing PCa from normal tissue. Preliminary results turned out that MTT, Vp, and PS from DP, Ktrans from ETM and Tofts, E, and PS from ATH, IAUC parameters and F from Brix could be helpful in discriminating prostate malignant lesions from prostate normal tissues. Parameter values from DP are correlated with those from ETM.

## Data Availability

The original contributions presented in the study are included in the article/supplementary material. Further inquiries can be directed to the corresponding authors.
